# The Stability of Model Human Coronaviruses on Textiles in the Environment and during Health Care Laundering

**DOI:** 10.1128/mSphere.00316-21

**Published:** 2021-04-28

**Authors:** Lucy Owen, Maitreyi Shivkumar, Katie Laird

**Affiliations:** a Infectious Disease Research Group, The Leicester School of Pharmacy, De Montfort University, Leicester, United Kingdom; Emory University School of Medicine

**Keywords:** TCID_50_, coronavirus, health care, human coronavirus OC43, infectivity, laundry, textile

## Abstract

Severe acute respiratory syndrome coronavirus 2 (SARS-CoV-2) persists on stainless steel and plastic for up to 7 days, suggesting that coronavirus disease 2019 (COVID-19) could be spread by fomite transmission. There is limited research on the stability of SARS-CoV-2 on textiles, with the risk of textiles acting as fomites not being well understood. To date, there does not appear to be any published research on the stability of coronaviruses during laundering, which is required to determine the efficacy of current laundering policies in the decontamination of health care textiles. The aim of this study was to investigate the environmental stability of human coronaviruses HCoV-OC43 and HCoV-229E on different textile fiber types and the persistence of HCoV-OC43 on textiles during domestic and industrial laundering. This study demonstrated that human coronaviruses (5 log_10_ 50% tissue culture infective doses [TCID_50_]) remain infectious on polyester for ≥72 h, cotton for ≥24 h, and polycotton for ≥6 h; HCoV-OC43 was also able to transfer from polyester to PVC or polyester after 72 h. Under clean conditions, HCoV-OC43 was not detectable on cotton swatches laundered with industrial and domestic wash cycles without temperature and detergent (≥4.57-log_10_-TCID_50_ reduction), suggesting that the dilution and agitation of wash cycles are sufficient to remove human coronaviruses from textiles. In the presence of interfering substances (artificial saliva), ≤1.78 log_10_ TCID_50_ HCoV-OC43 was detected after washing domestically without temperature and detergent, unlike industrial laundering, where the virus was completely removed. However, no infectious HCoV-OC43 was detected when washed domestically with detergent.

**IMPORTANCE** Synthetic textiles such as polyester could potentially act as fomites of human coronaviruses, indicating the importance of infection control procedures during handling of contaminated textiles prior to laundering. This study provides novel evidence that human coronaviruses can persist on textiles for up to 3 days and are readily transferred from polyester textile to other surfaces after 72 h of incubation. This is of particular importance for the domestic laundering of contaminated textiles such as health care uniforms in the United Kingdom and United States, where there may be a risk of cross-contaminating the domestic environment. It was demonstrated that human coronaviruses are removed from contaminated textiles by typical domestic and commercial wash cycles, even at low temperatures without detergent, indicating that current health care laundering policies are likely sufficient in the decontamination of SARS-CoV-2 from textiles.

## Abstract (precis)

Synthetic textiles such as polyester could potentially act as fomites of human coronaviruses, indicating the importance of infection control procedures during handling of contaminated textiles prior to laundering. This study provides novel evidence that human coronaviruses can persist on textiles for up to 3 days and are readily transferred from polyester textile to other surfaces after 72 h of incubation.

## INTRODUCTION

Severe acute respiratory syndrome coronavirus 2 (SARS-CoV-2), the causative agent of coronavirus disease 2019 (COVID-19), is primarily spread through respiratory droplets and/or aerosols (1). Fomite transmission is also considered a likely mode of infection (2) due to the detection of SARS-CoV-2 RNA on surfaces surrounding infected patients (3, 4) and the environmental stability of infectious SARS-CoV-2 *in vitro* (2, 5–7). This is supported by preliminary animal model evidence from the work of Sia et al. (8), where it was reported that one in three golden Syrian hamsters exposed to housing contaminated with SARS-CoV-2 began to shed infectious virus from 1 day postcontact, although fomite transmission was less efficient than by direct contact and aerosol transmission (8). Enhanced disinfection and hand hygiene procedures have been widely implemented in health care and community settings to limit the potential risk of fomite transmission (9).

Published research on the persistence of SARS-CoV-2 has primarily focused on nonporous surfaces. For example, van Doremalen et al. (7) reported that SARS-CoV-2 remained infectious on plastic and stainless steel for up to 72 h at room temperature, where a 50% tissue culture infective dose (TCID_50_)/ml of 10^0.6^ was reported, and Chin et al. (5) concluded that SARS-CoV-2 persisted for 7 days on stainless steel and plastic. There is limited research in the published literature on the persistence of SARS-CoV-2 on a range of textiles, and the potential for fomite transmission from textiles is not well defined. SARS-CoV-2 appears to be less stable on porous surfaces than on nonporous surfaces, suggesting that SARS-CoV-2 may persist to a lesser extent on some textiles; for example, SARS-CoV-2 was detected up to 7 days postinoculation on cotton, compared to 28 days on nonporous surfaces (2). The infectious viral titer of SARS-CoV-2 was shown to decrease more rapidly on porous surfaces (including cotton and respirator masks) than on nonporous surfaces (nitrile gloves, stainless steel, and plastic) (10). Similarly, SARS-CoV-2 remained infectious on paper for 3 h and cloth and wood for 2 days, in contrast to recovery of infectious virus on nonporous surfaces (plastic, stainless steel, and surgical masks) ranging from 4 to 7 days (5).

The properties of textiles can vary widely depending on the fiber type (e.g., synthetic or natural fibers) and construction of the material (e.g., woven versus nonwoven textiles), which can impact the persistence and transfer efficiency of microorganisms. For example, the transfer efficiency of bacteria was greater from woven viscose and polyester than from cotton, silk, and polypropylene (11). Increasing our understanding of the potential risk of SARS-CoV-2 persistence on textiles is of particular importance in health care settings to inform infection control policies for handling used linen and staff uniforms before laundering in the domestic, care home, and hospital environments (12).

There does not appear to be any research on the stability of SARS-CoV-2 on textiles during laundering, with a need to determine the efficacy of current laundering processes in removing SARS-CoV-2 from textiles and preventing cross-contamination to other textiles in the wash. Industrial laundering of health care and commercial linen is typically conducted under thermal decontamination conditions (≥60°C) or at lower temperatures with the addition of disinfectants (13). In the United States, the Centers for Disease Control and Prevention (CDC) state that thermal disinfection should be conducted at 71°C for a minimum of 25 min (14), whereas in the United Kingdom, 65°C for ≥10 min or 71°C for ≥3 min is required for thermal disinfection (15). However, in both the United Kingdom and United States, there are no additional recommendations for enhanced decontamination procedures within infection control guidelines during the COVID-19 pandemic (16, 17). In the United Kingdom, health care workers are generally required to launder their uniforms at home, where policies state that they should be washed at the highest temperature suitable for the textile and separated from household laundry (18). A disadvantage of domestic laundering is the inability to validate and monitor the decontamination efficacy, which may differ between households due to factors such as variation in domestic washing machine performance and lack of adherence to laundering policies. Investigating the persistence of coronaviruses during laundering would enhance understanding of the potential risk of textiles as fomites for SARS-CoV-2 and suggest whether there are any requirements for reconsideration of health care laundry policies.

Another disadvantage of domestic laundering is that handling contaminated uniforms prior to laundering may lead to cross-contamination of other surfaces within the household. The National Health Service (NHS) England uniform policy does not specify how uniforms should be handled (18), in contrast to industrial laundering, where infection control procedures are in place for handling of infectious linen (12). In response to the COVID-19 pandemic, some NHS staff adopted the practice of transporting their worn uniforms home in cloth laundry bags or pillowcases and placing these directly in the washing machine for laundering (19), reducing the need for handling of contaminated clothing and the subsequent risk of contaminating surfaces while traveling home or within the home itself. Investigating the efficacy of laundering within cloth bags for the removal of coronaviruses is therefore warranted.

Human coronaviruses HCoV-OC43 and HCoV-229E were used in this study as model organisms for SARS-CoV-2 to investigate the environmental persistence of coronaviruses on textiles and during laundering. HCoV-OC43, HCoV-229E, and SARS-CoV-2 are members of the *Coronaviridae* family of viruses, with HCoV-OC43 and SARS-CoV-2 belonging to the *Betacoronavirus* genus and HCoV-229E to the *Alphacoronavirus* genus (20, 21). The general structure and organization of viruses in the *Coronaviridae* family are similar; they are single-stranded positive-sense RNA viruses with a lipid envelope and spike proteins projecting from their surface (22), indicating that the stability of SARS-CoV-2 may be similar to that of HCoV-OC43 and HCoV-229E. In accordance, a literature review by Aboubakr et al. (23) indicated that the environmental persistence of coronaviruses is comparable (23).

The aim of this study was to investigate the environmental stability of HCoV-OC43 and HCoV-229E on different textile fiber types and their persistence on textiles during domestic and industrial laundering. The infectious viral titer of HCoV-OC43 and HCoV-229E was measured as a means to infer the potential risk of fomite transmission from textile surfaces, in contrast to the detection of viral RNA, which does not distinguish between infectious and inactive virus particles.

## RESULTS

### Development of methodologies to recover coronaviruses from textiles.

**FIG 1:**
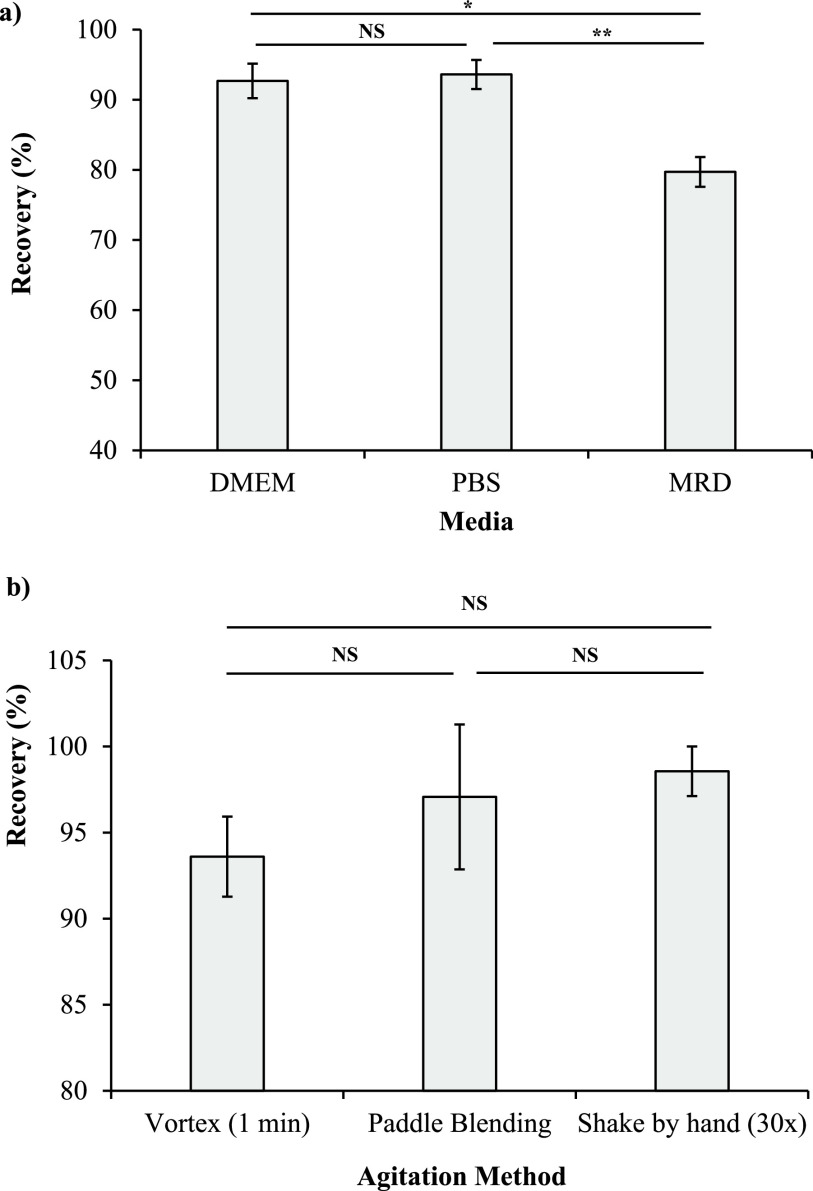
Percent recovery of HCoV-OC43 from 100% cotton with different recovery media (a) and different agitation methods (b) (mean, *n* = 3 ± SEM). Inoculum, 5 log_10_ TCID_50_/25 cm^2^. Significance of differences was determined using an ANOVA with Tukey’s multiple comparisons. *, *P* ≤ 0.05; **, *P* ≤ 0.01; NS, not significant (*P* > 0.05).

The recovery of HCoV-OC43 from textiles was optimized by testing a range of media and agitation methods. The recovery of HCoV-OC43 from 100% cotton was not significantly different (*P* ≤ 0.05; analysis of variance [ANOVA] with Tukey’s multiple comparisons) after vortexing for 1 min with Dulbecco’s modified Eagle’s medium (DMEM; 92.68%) or phosphate-buffered saline (PBS; 93.63%) (Fig. 1a). Both DMEM and PBS had a significantly greater (*P* ≤ 0.05) recovery efficiency than maximum recovery diluent (MRD; 79.71%). PBS was carried forward for future experiments due to being a more economical alternative to DMEM. The recovery efficiency of HCoV-OC43 from cotton was not significantly different (*P* > 0.05; ANOVA with Tukey’s multiple comparisons) between the agitation methods tested: vortexing for 1 min, paddle blending at 230 rpm for 1 min, and shaking by hand 30 times (Fig. 1b). Shaking by hand was selected as the recovery method for further testing due to the highest mean recovery efficiency (98.56%) and greatest precision (standard error of the mean [SEM] = 1.04%) of the three methods. An advantage of the shaking-by-hand method is that no specialized equipment is required, and therefore, this could be employed across industrial laundries to allow for standardization of recovery methods.

### Stability of infectious HCoV-OC43 and HCoV-229E on textiles.

**FIG 2:**
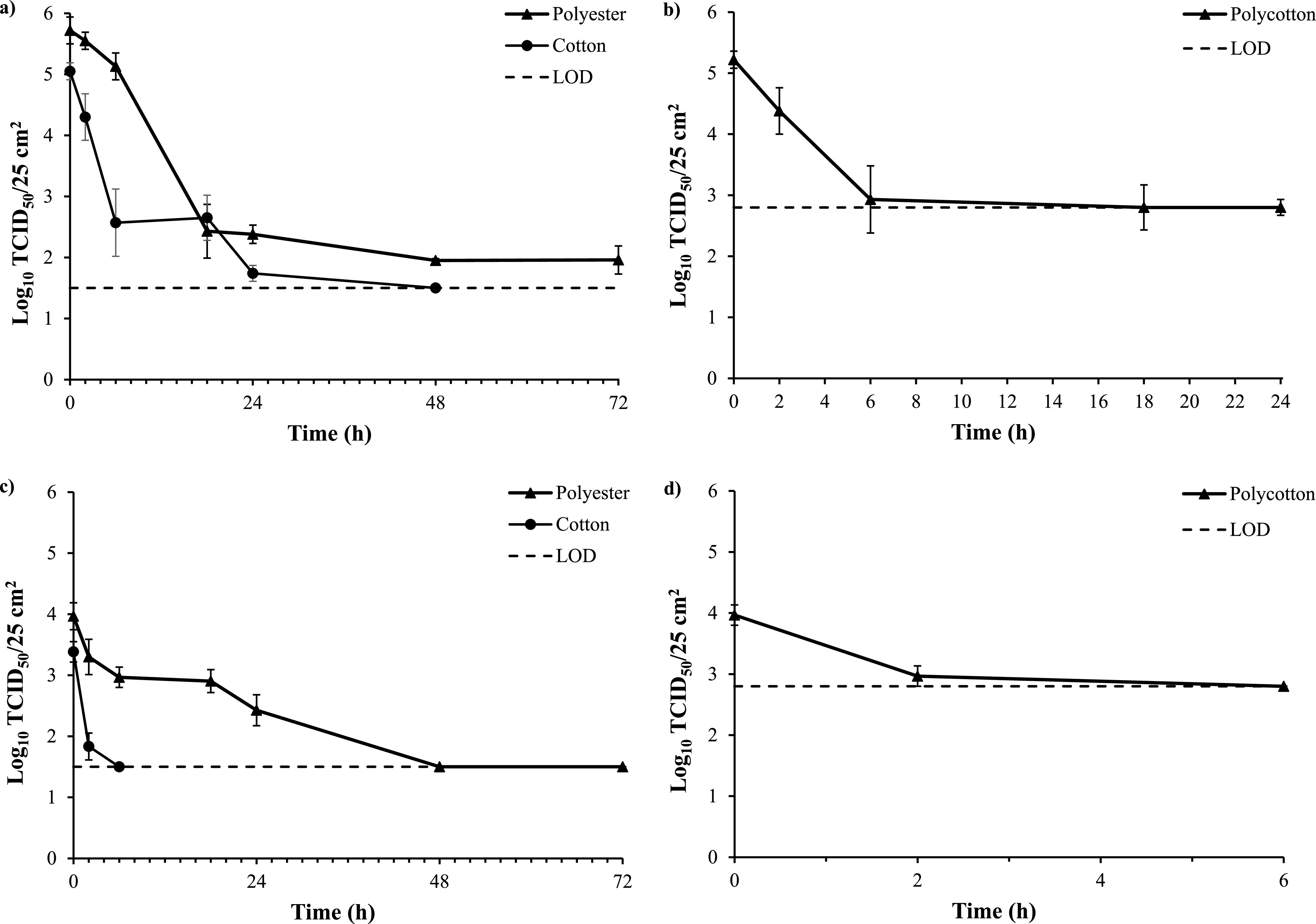
Stability (log_10_ TCID_50_/25 cm^2^) of HCoV-OC43 (a and b) and HCoV-229E (c and d) on textiles over time at ambient temperature (mean, *n* = 3 ± SEM). Inoculum, 5 log_10_ TCID_50_/25 cm^2^.

The infectivity of HCoV-OC43 (5 log_10_ TCID_50_/25 cm^2^) decreased over time on 100% cotton, 99.3% polyester, and 65%/35% polyester-cotton blend (polycotton) at ambient temperatures (19 ± 0.5°C and 34% ± 2% relative humidity; Fig. 2a and b). HCoV-OC43 was the most stable on polyester, remaining infectious for at least 72 h, where 1.96 log_10_ TCID_50_/25-cm^2^ textile sample was detected (Fig. 2a). HCoV-OC43 was detectable on cotton for 24 h, where 1.7 log_10_ TCID_50_/25 cm^2^ was detected, before decreasing below the limit of detection (LOD) of 1.5 log_10_ TCID_50_/25 cm^2^ (Fig. 2a). Polycotton produced a cytotoxic effect against mammalian cells, reducing the LOD of the assay to 2.80 log_10_ TCID_50_/25 cm^2^. The infectivity of HCoV-OC43 on polycotton was reduced at a similar rate as cotton, from 5.22 to 2.93 log_10_ TCID_50_/25 cm^2^ within 6 h, before reaching the LOD within 18 h (Fig. 2b). No infectious virus (≤1.5 ± 0.0 log_10_ TCID_50_/25 cm^2^) was detected on the surface of petri dishes housing the inoculated swatches of cotton and polycotton after 0, 6, and 24 h of contact, demonstrating that the stability of infectious virus was not due to leaching onto the plastic surface. Polyester was not absorbent, and therefore leaching of the inoculum onto the petri dish was not observed.

HCoV-229E was less stable than HCoV-OC43, remaining detectable for 24 h on polyester (Fig. 2c) and less than 6 h on cotton (Fig. 2c) and polycotton (Fig. 2d). HCoV-OC43 was used for all subsequent investigations due to its greater environmental stability compared to HCoV-229E.

### Wet/dry attachment of HCoV-OC43 to textile fibers.

**FIG 3:**
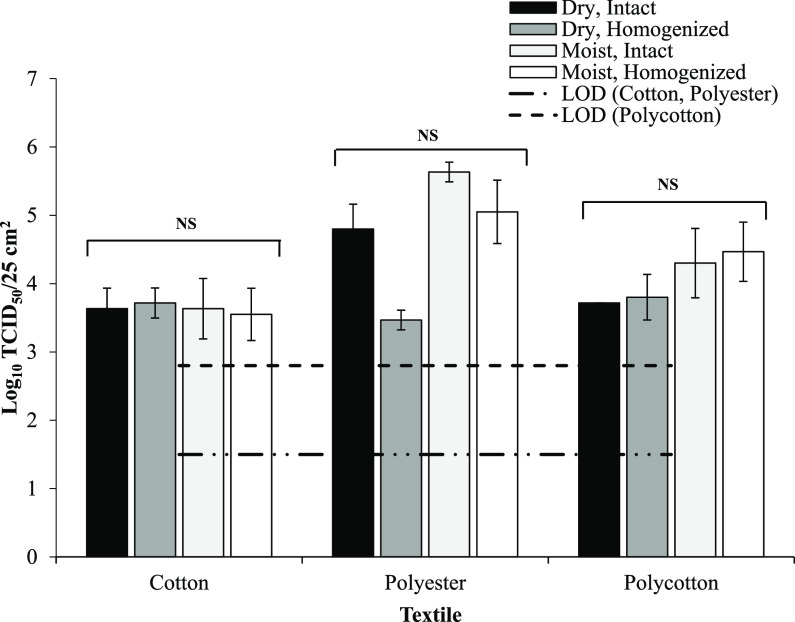
Log_10_ TCID_50_/25-cm^2^ recovery of HCoV-OC43 from wet and dry intact and homogenized textile samples (mean, *n* = 3 ± SEM). Inoculum, 5 log_10_ TCID_50_/25 cm^2^. Significance of differences was determined using the independent-sample Kruskal-Wallis test. NS, not significant (*P* ≥ 0.05).

The recovery of HCoV-OC43 from intact textile samples was compared to that of textile samples that were homogenized, to investigate if the reduction in viral infectivity observed over time on textiles was the result of HCoV-OC43 attachment to textile fibers or entrapment within the weave of the textiles. There was no significant difference (*P* > 0.05; independent-sample Kruskal-Wallis test) in recovery of HCoV-OC43 between intact and homogenized polycotton, cotton, or polyester samples (Fig. 3), suggesting that HCoV-OC43 does not attach to textile fibers or become entrapped within the weave of the textile. The recovery of HCoV-OC43 was also compared between dry and premoistened textiles to investigate if there was absorption of virus within the textile fiber. There was no significant difference (*P* > 0.05; independent-sample Kruskal-Wallis test) in the recovery of HCoV-OC43 from dry and premoistened textile samples (Fig. 3).

### Transfer of HCoV-OC43 from textiles to other surfaces.

The transfer of infectious HCoV-OC43 from textile samples to polyvinyl chloride (PVC) plastic and another textile were investigated both immediately after inoculation and at the maximum time where HCoV-OC43 was detectable for each textile (polycotton, 2 h; cotton, 18 h; polyester, 72 h; Fig. 2a and b). HCoV-OC43 transfer was detected from polyester onto PVC and polyester samples up to 72 h postinoculation (Table 1). No transfer from cotton or polycotton was detected immediately after inoculation and after 2 to 18 h of incubation at room temperature, where ≥2.63 log_10_ TCID_50_/25 cm^2^ was detected on the donor textile (Table 1). There was no significant difference (*P* > 0.05; independent-sample Kruskal-Wallis test) in the titer of infectious virus transferred to PVC and textile for all textiles at all time points.

**TABLE 1:** Log_10_ TCID_50_/25cm^2^ transfer of HCoV-OC43 from textiles to PVC PUR or the same textile fiber type over time^*a*^

Textile	Time (h)	Transfer material		
		Donor (pretransfer)	PVC PUR (recipient)	Textile (recipient)
Cotton	0	4.36 ± 0.54	*^b^	*
	18	3.07 ± 0.08	*	*

Polycotton	0	4.72 ± 0.08	*	*
	2	2.63 ± 0.14	*	*

Polyester	0	5.24 ± 0.52	2.28 ± 0.39	2.38 ± 0.34
	24	3.55 ± 0.71	2.03 ± 0.03	2.61 ± 0.59
	72	2.50 ± 0.54	1.64 ± 0.14	1.67 ± 0.17

a Data shown as mean ± SEM (*n* = 3). Inoculum, 5 log_10_ TCID_50_/25 cm^2^.

b Asterisk indicates value below detection limit (2.45 log_10_ TCID_50_**/**25 cm^2^ for polycotton; 1.5 log_10_ TCID_50_/25 cm^2^ for polyester, cotton, and PVC PUR). No significant differences (*P *≥ 0.05) in infectious virus transferred to PVC and textile were determined according to the independent-sample Kruskal-Wallis test.

### Removal of HCoV-OC43 from textiles with domestic, industrial, and OPL wash cycles.

The persistence of HCoV-OC43 on cotton swatches following laundering using domestic (40°C), industrial (67°C), and on-premises laundering (OPL) (75°C) wash cycles, with and without detergent and temperature, was investigated. Wash cycle parameters used are displayed in Table 2. HCoV-OC43 (8 log_10_ TCID_50_/25 cm^2^) was inoculated onto cotton in the presence of either DMEM culture medium or artificial saliva as interfering substances.

**TABLE 2:** Domestic, industrial, and on-premises laundering cycle parameter investigated^*a*^

Stage	Domestic		Industrial		On-premises laundering (OPL)	
	Temp (°C)	Hold time (min)	Temp (°C)	Hold time (min)	Temp (°C)	Hold time (min)
Prewash	—^*b*^		35 (32.18 ± 0.16)	3 (4 ± 0.4)	40 (40.50 ± 0.22)	4 (3 ± 0.0)
Main wash	40 (39.55 ± 0.05)	21 (23 ± 0.77)	67 (61.79 ± 0.08)	10 (11.6 ± 0.1)	75 (68.08 ± 0.06)	10 (13 ± 0.5)
Rinse/spin	Cold input	44	Cold input	11	Cold input	15
Cycle duration (min)		95		85		96

a Measured mean peak temperatures and holding times are shown in parentheses (*n* ≥ 4 independent washes; mean ± SEM).

b —, not applicable.

In the presence of DMEM, no HCoV-OC43 was recovered from cotton swatches following domestic, industrial, or on-premises laundering without heat or detergent (≤1.5 log_10_ TCID_50_/25 cm^2^; Table 3).

**TABLE 3:** Log_10_ TCID_50_/25-cm^2^ recovery of infectious HCoV-OC43 from 100% cotton following domestic 40°C, industrial 67°C, and OPL 75°C wash cycles with and without temperature and detergent^*a*^

Condition	Log_10_ TCID_50_/25 cm^2^^*b*^ (log_10_ reduction^*c*^)							
	Domestic		Domestic (swatch in pillowcase)		Industrial^*d*^		OPL^*d*^	
	DMEM	Artificial saliva	DMEM	Artificial saliva	DMEM	Artificial saliva	DMEM	Artificial saliva
Ambient temp, no detergent	≤1.5 ± 0.00 (≥4.57)	≤1.78 ± 0.19 (≥4.52)	—^*e*^	≤1.54 ± 0.04 (≥4.76)	≤1.5 ± 0.00 (≥4.57)	≤1.5 ± 0.00 (≥4.80)	≤1.5 ± 0.00 (≥4.57)	≤1.5 ± 0.00 (≥4.80)
Ambient temp + detergent	≤1.5 ± 0.00 (≥4.57)	≤1.5 ± 0.00 (≥4.80)	—	≤1.5 ± 0.00 (≥4.80)	—	—	—	—
Temp + detergent	≤1.5 ± 0.00 (≥4.57)	≤1.5 ± 0.00 (≥4.80)	—	≤1.5 ± 0.00 (≥4.80)	—	≤1.5 ± 0.00 (≥4.80)	—	≤1.5 ± 0.00 (≥4.80)

a *n* = 6 ± SEM. Inoculum, 8 log_10_ TCID_50_/25 cm^2^.

b The detection limit was 1.5 log_10_ TCID_50_/25 cm^2^. Where one or more samples reached the detection limit, the number of infectious virus is expressed as ≤log_10_ TCID_50_/25 cm^2^.

c Log_10_ reduction calculated from initial viral load on swatch; DMEM, 6.07 log_10_ TCID_50_/25 cm^2^; artificial saliva, 6.30 log_10_ TCID_50_/25 cm^2^.

d Full industrial and OPL wash systems (temperature plus detergent) were tested against HCoV-OC43 in the presence of artificial saliva to confirm the removal of HCoV-OC43 by typical in-use conditions. However, the individual parameters of temperature and detergent were not tested, due to a lack of detectable virus on textiles when washed with water alone, preventing any effect of the detergent and temperature parameters above that of water alone from being detected.

e —, not tested.

Where HCoV-OC43 was applied to cotton in artificial saliva, 1.78 ± 0.19 log_10_ TCID_50_/25 cm^2^ HCoV-OC43 was recovered after domestic laundering without heat and detergent (removal via dilution and agitation alone). However, no infectious virus was recovered (≤1.5 log_10_ TCID_50_/25 cm^2^) from cotton following domestic washing at ambient temperature (23.44 ± 0.06°C) or 40°C with detergent (Table 3). In addition, infectious virus was not detected after industrial and OPL cycles with or without heat and detergent (Table 3).

There was no significant difference (*P* > 0.05; Mann-Whitney U test) in recovery of infectious virus from cotton after a domestic ambient wash enclosed within a pillowcase (≤1.54 ± 0.04 log_10_ TCID_50_/25 cm^2^) compared to cotton that was loose in the wash.

No infectious virus was detected on sterile swatches placed in the wash for any conditions tested (≤1.5 log_10_ TCID_50_/25 cm^2^), indicating that detectable levels of cross-contamination did not occur. The no-virus controls did not recover any infectious virus (≤1.5 log_10_ TCID_50_/25 cm^2^).

### Moist heat thermotolerance of HCoV-OC43.

**FIG 4:**
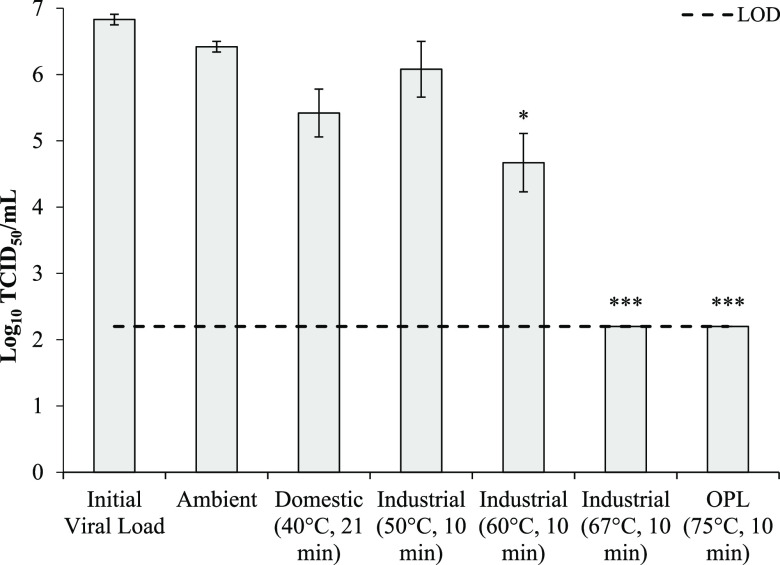
Stability of HCoV-OC43 (log_10_ TCID_50_/ml) in water following treatment with typical domestic, industrial, and OPL wash temperature cycles as described in Table 2 (mean, *n* = 3 ± SEM). Inoculum, 8 log_10_ TCID_50_/ml. Significance of differences from the initial viral load was determined using the independent-sample Kruskal-Wallis test. *, *P* ≤ 0.05; **, *P* ≤ 0.01; ***, *P* ≤ 0.005.

Due to HCoV-OC43 being largely removed from cotton during the domestic, industrial, and OPL wash cycles without detergent, the stability of infectious HCoV-OC43 in water and moist-heat thermotolerance were investigated in a closed system to remove the effects of dilution and agitation that apply during laundering. The temperature-time relationship was based upon the wash parameters, where 40°C for 21 min or 67°C and 75°C for 10 min were used. Industrial wash parameters were also performed at 50°C and 60°C for 10 min to further investigate the thermotolerance of HCoV-OC43, representing typical industrial laundering temperatures for the hospitality sector. HCoV-OC43 was stable in water at ambient temperatures for 95 min, where a 0.42-log_10_ reduction from the initial viral load of 6.83 log_10_ TCID_50_/ml was observed (Fig. 4). The infectivity of HCoV-OC43 was not significantly reduced (*P* > 0.05) by exposure to the 40°C domestic wash cycle temperatures (40°C, 21 min). Exposure to industrial thermal disinfection temperatures of 67°C and 75°C for 10 min reduced HCoV-OC43 to below the detection limit of 2.2 log_10_ TCID_50_/ml. HCoV-OC43 was not significantly reduced (*P* > 0.05) by exposure to the 50°C industrial wash parameters but was significantly (*P* ≤ 0.05) reduced after heating to 60°C for 10 min, with a reduction of 2.17 log_10_ TCID_50_/ml.

## DISCUSSION

The results of this study demonstrate that human coronaviruses can remain infectious on polyester for ≥72 h, cotton for ≥24 h, and polycotton for ≥6 h (Fig. 2), suggesting that, in addition to hard surfaces, textiles could also potentially act as fomites of SARS-CoV-2. The cytotoxicity of polycotton toward mammalian cell lines reduced the LOD of the assay against this textile, meaning that HCoV-OC43 and HCoV-229E were not detected (≤2.8 log_10_ TCID_50_/25 cm^2^) after only 6 h. Polycotton reduced at a similar rate as cotton up to 6 h against HCoV-OC43 and HCoV-229E, suggesting that the overall stability may be similar to cotton. The observed cytotoxicity could be related to treatments applied to the textile or residues from the manufacturing process (24). There may be variation in the effects of polycotton samples depending on the finishing treatments applied in addition to the ratio of polyester and cotton employed; further investigation of a wide range of polycotton samples may therefore be warranted in the future.

Overall, HCoV-OC43 and HCoV-229E were stable for greater periods of time on polyester compared to cotton and polycotton (Fig. 2), demonstrating the significant variation in persistence of coronaviruses on different textile fiber types. Polyester is a hydrophobic synthetic fiber and therefore has low moisture-absorbing properties; in contrast, cotton is a natural fiber that readily absorbs water (25). The results of this investigation are in accordance with those of Kasloff et al. (10), where SARS-CoV-2 persisted for less than 24 h on cotton and was more stable on synthetic materials from respirator masks (14 days) and nonporous surfaces such as plastic (21 days) and nitrile gloves (4 days). Other factors in the stability of coronaviruses on surfaces include temperature and relative humidity. SARS-CoV-2 degraded more rapidly on nonporous surfaces at 35°C than 24 to 28°C and at a relative humidity of 20 to 40% compared to 60% (26). In this study, investigations were performed at 19.0 ± 0.5°C and 34% ± 2% relative humidity; the stability profile may differ to some extent within different climates.

The recovery of HCoV-OC43 from textiles was not increased by premoistening the textile, and therefore limiting absorption of the viral inoculum, suggesting that HCoV-OC43 was not absorbed into the textile fibers. Moreover, homogenization of textile samples did not increase recovery of HCoV-OC43, suggesting that HCoV-OC43 was not attached to the textile fibers. It is speculated that the more rapid viral inactivation on natural fibers could be associated with desiccation of the virus following absorption of water by the textile fibers; coronaviruses possess a lipid envelope which is susceptible to desiccation, thereby leading to inactivation (27).

The stability of HCoV-229E has been more extensively reported in the published literature and is reported to be comparable to that of other coronaviruses such as SARS-CoV (28). HCoV-OC43 was more stable on textiles than HCoV-229E; in contrast, previous research concluded that HCoV-229E was inactivated on cotton gauze sponge within 12 h, compared to 3 h for HCoV-OC43 (28). The stability of HCoV-OC43 may be more comparable than HCoV-229E to that of SARS-CoV-2, where previous research has demonstrated persistence of SARS-CoV-2 on cotton for 7 days (2) and cloth of an unspecified fiber type for 2 days (5).

For an object to act as a fomite, it must be able to transfer microorganisms onto human skin or other surfaces/objects; however, there do not appear to be any studies in the published literature on the transfer of SARS-CoV-2 or other coronaviruses from textiles onto other surfaces. Here, we demonstrated that HCoV-OC43 was able to transfer from polyester textile onto PVC and other fabric samples up to 72 h postinoculation, whereas no transfer was detected from cotton or polycotton immediately after inoculation (Table 1). Previous research has demonstrated that bacteria transfer more efficiently from polyester than cotton and polycotton. The magnitude of bacterial transfer was inversely correlated with surface roughness, with cotton and polyester being rougher than polyester. The presence of moisture was an important factor in transfer of bacteria between textiles (11). The transfer of HCoV-OC43 from polyester in this study could be attributed to polyester’s low porosity, allowing moisture from the viral inoculum to remain on the surface of the textile. In contrast, polycotton and cotton have a higher moisture regain, leaving less moisture available at the surface for viral transfer (11, 33). Absorption of the viral inoculum into the textile could also limit transfer with only virions remaining on the outer surface of the textile coming into contact with the transfer surface. These findings are of particular importance for the laundering of polyester health care garments, for example, surgical gowns. Health care uniforms are typically comprised of polycotton, suggesting that the risk of coronaviruses transferring from contaminated uniforms to surfaces within the home environment is lower than that for polyester.

There does not appear to be any research on the stability of SARS-CoV-2 or other coronaviruses on textiles during laundering. The persistence of SARS-CoV-2 on laundered textiles could lead to cross-contamination of other textiles within the wash and pose a risk of transmission to the end user; this is of particular concern within the health care sector, where patients are clinically vulnerable (12). In this study, the stability of HCoV-OC43 on cotton during laundering was investigated. Cotton is a common material within the health care sector for items such as bed linens. Polycotton and polyester were not investigated during the wash due to polycotton being cytotoxic toward BHK-21 cells, resulting in a reduced LOD of the assay (impacting the ability to calculate reductions in the wash), while polyester was hydrophobic and therefore it is hypothesized that HCoV-OC43 would be readily removed.

Laundering for the removal of microorganisms from textiles relies on a number of factors including temperature, agitation, dilution in water, and the use of detergents (13). In the absence of interfering substances (artificial saliva), domestic laundering with and without temperature and detergent removed infectious HCoV-OC43 from cotton (≤1.5 log_10_ TCID_50_/25 cm^2^; Table 3). This suggests that the dilution and agitation during laundering are sufficient to remove detectable levels of HCoV-OC43 from the textile. Conversely, in the presence of artificial saliva, HCoV-OC43 was detected at low levels (≤1.78 log_10_ TCID_50_/25 cm^2^) following a domestic wash cycle without temperature and detergent. The presence of protein within artificial saliva, in the form of mucin, may act as an interfering substance that reduces the removal of HCoV-OC43 from the textile during laundering; in accordance, Bockmuhl et al. (13) stated that the presence of soiling influences the decontamination of textiles. However, no infectious virus was detected on cotton after domestic washing with detergent, both at ambient temperature and at 40°C (Table 3). Laundry detergents contain surfactants that aid in the removal of soiling and microorganisms from textiles, in addition to antimicrobial agents such as active oxygen bleach (13). Detergents have also been shown to inactivate coronaviruses due to the disruption of the lipid envelope; for example, SARS-CoV-2 was reduced by 5.7 to 6.5 log_10_ TCID_50_ using 0.1 to 0.5% sodium dodecyl sulfate (29).

Infectious HCoV-OC43 (with and without artificial saliva) was not detectable on cotton after laundering in a commercial washer-extractor machine using industrial and OPL wash cycles without temperature and detergent (Table 3). These results suggest that the dilution and agitation effect upon the removal of HCoV-OC43 was greater within the commercial washer-extractor machine than within the domestic washer-extractor when interfering substances were present. The effects of detergent alone were not determined within the industrial and OPL cycles due to the lack of detectable HCoV-OC43 after washing with cold water alone; however, the full wash system of detergent and elevated temperatures (67 to 75°C) was tested to confirm the behavior of HCoV-OC43 under typical in-use conditions for the sector. Indeed, no HCoV-OC43 was detected following laundering using the industrial or OPL wash cycles with temperature and detergent (Table 3).

During the COVID-19 pandemic, some UK NHS staff have transported worn uniforms home in cloth laundry bags or pillowcases, which are placed directly in the washing machine (19). This method may reduce the handling of contaminated uniforms and subsequent risk of contaminating the domestic environment. In a similar manner, infected linens are placed within water-soluble bags prior to OPL or industrial laundering (15). The efficacy of domestic laundering within cloth bags was investigated by enclosing HCoV-OC43-inoculated cotton swatches within a pillowcase; it was hypothesized that this could reduce the agitation and dilution of the textiles enclosed within a bag. However, no infectious HCoV-OC43 was observed in the presence of artificial saliva after laundering with detergent in ambient or warm (40°C) water. The use of cloth laundry bags or pillowcases to contain uniforms could therefore be a useful strategy to reduce the need for handling of contaminated uniforms within the home environment.

The results of this investigation demonstrate that both domestic and industrial laundering reduce HCoV-OC43 contamination on textiles in the presence of artificial saliva by ≥4.52 to 4.80 log_10_ TCID_50_/25 cm^2^ without the use of detergent and thermal disinfection temperatures. A 5-log_10_ reduction of bacteria is considered to demonstrate disinfection efficacy of laundering processes according to health care laundry policies in the United Kingdom (30) and Germany (31), suggesting that decontamination was likely achieved (considering the LOD of the assay) simply by washing with water alone. Overall, these data suggest that laundering under current policies and guidelines is likely to be sufficient in the sanitization of textiles contaminated with coronaviruses.

The removal of microorganisms from contaminated textiles during laundering may lead to cross-contamination of other textiles within the wash, specifically where microorganisms are not inactivated by the wash parameters (temperatures, detergents, and/or disinfectants) employed. Similar investigations using bacteria have demonstrated cross-contamination of other textiles within a wash cycle. Tarrant et al. (32) recovered 2.72 to 2.89 log_10_ CFU Clostridioides difficile spores from previously sterile textiles after industrial laundering alongside swatches contaminated with 7 log_10_ CFU C. difficile spores (32). Riley et al. (33) recovered 3.05 to 3.23 log_10_ CFU Escherichia coli and Staphylococcus aureus from previously sterile swatches laundered domestically at 40°C with detergent (33). In this study, no cross-contamination of HCoV-OC43 was detected during all wash cycles, suggesting that HCoV-OC43 is either inactivated during the washing conditions or diluted to below the detection limit of the assay employed (1.5 log_10_ TCID_50_/25 cm^2^). The results suggest that it is unlikely for textiles to become significantly contaminated with infectious coronaviruses during laundering and that the risk of this is lower than for bacteria.

To investigate if human coronaviruses could remain infectious within the wash after being removed from contaminated textiles, the stability of HCoV-OC43 in water at standard domestic and commercial washing temperatures was investigated. SARS-CoV-2 has been reported to remain infectious for a least 7 days in tap water at ambient temperature but was sensitive to temperature, reducing by approximately 3 log_10_ TCID_50_/ml within 1 h at 50°C, and approximately 4 log_10_ TCID_50_/ml within 10 min at 70°C (34), suggesting that SARS-CoV-2 could persist during laundering at lower temperatures. In accordance with the findings of Bivins et al. (34), HCoV-OC43 was stable in tap water both at ambient temperature for the time duration of a typical domestic wash cycle (95 min) and under the conditions of a simulated 40°C domestic wash cycle (peak temperature 40°C, 21 min) and 50°C industrial laundering cycle (peak temperature 50°C, 10 min; Fig. 4). In contrast, a complete (≥4.63 log_10_ TCID_50_/ml) reduction in HCoV-OC43 was achieved by exposure to 67 to 71°C for 10 min. These results are in accordance with routine thermal disinfection parameters of 71°C for ≥25 min outlined by the U.S. CDC (14) and of 65°C for ≥10 min or 71°C for ≥3 min outlined by the UK Department of Health (15). Notably, HCoV-OC43 was reduced by only 2.17 log_10_ TCID_50_/ml after 60°C for 10 min, which is considered a standard time and temperature for industrial laundering in the hospitality sector. This demonstrates the importance of the overall wash process in achieving textile decontamination, including dilution, agitation, detergents, and disinfectants (13). Overall, these results suggest that human coronaviruses can remains infectious in low-temperature wash water following removal from textiles; however, the load of infectious virus being deposited onto other textiles in the wash is likely to be low (Table 3) due to dilution within the volume of water (main wash stage volumes = 4.4 liters, domestic; 16 liters, industrial and OPL). Detergents could also inactivate virus within the wash water.

In addition to the laundering process, the load of microorganisms present on textiles may be further reduced during drying. In one published study, the total viable count of bacteria on scrub suit materials was >10 log_10_ CFU/ml after a 40°C wash cycle; however, this was further reduced to 9.28 log_10_ CFU/ml upon air drying, 1.70 log_10_ CFU/ml by air drying and ironing, and 2.52 log_10_ CFU/ml by tumble drying (35). Drying practices can vary significantly; for health care workers laundering their uniforms domestically, there do not appear to be set parameters for air drying or tumble drying (18). The time or temperature of drying will also vary based on the textile fiber being dried (14). In industrial laundries, textiles may be dried using tumble driers or pressed and dried simultaneously using high temperatures and pressures such as 175°C with 400 kPa of pressure for 3 s (32). Coronaviruses may also be reduced by drying practices; however, this was not investigated due to the removal of detectable HCoV-OC43 by laundering alone.

In this study, the viral load applied to textiles was 5 log_10_ TCID_50_/25 cm^2^ for survival experiments and 8 log_10_ TCID_50_/25 cm^2^ for laundering investigations. This is in line with similar published studies (2, 5–7). It has been suggested that the use of high viral loads does not reflect those found within real-life settings. The infectious dose of SARS-CoV-2 is not well understood (36), making it difficult to ascertain the viral load required for contaminated surfaces to act as fomites. Moreover, the load of infectious SARS-CoV-2 shed onto environmental surfaces has yet to be reported in the published literature, with studies focusing on the detection of viral RNA (3, 4), not allowing for distinction between infectious and noninfectious virus. Conversely, a high viral titer was used in this study to enable the log_10_ reduction in viral load to be calculated above the limit of detection for the assay, thereby determining the overall sensitivity of the virus to the environmental conditions under a worst-case scenario.

This investigation provides evidence to suggest that typical domestic and industrial wash programs are capable of removing high titers of human coronaviruses from contaminated textiles and preventing significant levels of cross-contamination to other textiles within the wash. As a result, textiles laundered in accordance with current health care laundering policies are likely to be sufficiently decontaminated without the need for enhanced decontamination processes. However, HCoV-OC43 was demonstrated to persist for a number of days on dry textiles and is capable of transferring to other surfaces from polyester, demonstrating the importance of infection control procedures prior to laundering. It would be advantageous to employ verified contamination controls, which industrial processes are more equipped to achieve than at home or within on-premises laundry settings. Contamination controls should include soiled linen management, trained personnel, barrier segregation, and accurately calibrated thermal disinfection validation and chemical dosing. In a broader sense, industrial processes may also be a greener alternative because of the highly optimized energy usage and economy of scale.

## MATERIALS AND METHODS

### Cell lines.

All cell lines were cultured at 37°C with 5% CO_2_. HCT-8 epithelial cells (ECACC 90032006) were cultured in RPMI 1640 medium (Lonza, Basel, Switzerland) with 10% fetal bovine serum (FBS; HyClone, Logan, UT, USA) and 1% penicillin-streptomycin (100 IU/ml penicillin, 100 μg/ml streptomycin; Lonza, Basel, Switzerland). BHK-21 clone 13 (ECACC 85011433) and MRC-5 (ATCC CCL-171) fibroblast cells were cultured in DMEM (Lonza, Basel, Switzerland) with 10% FBS and 1% penicillin-streptomycin.

### Viruses.

HCoV-OC43 (ATCC VR-1558) was grown in HCT-8 cells in RPMI 1640 with 5% FBS and 1% penicillin-streptomycin (7 days, 33°C, 5% CO_2_). HCoV-229E (ATCC VR-740) was grown in MRC-5 fibroblast cells in DMEM with 5% FBS and 1% penicillin-streptomycin (4 days, 33°C, 5% CO_2_). Virus stocks were obtained by harvesting the medium from infected cells and centrifuging at 3,000 × *g* for 4 min to remove cell debris.

For laundering experiments, HCoV-OC43 was concentrated using polyethylene glycol (PEG) precipitation. A 4× PEG solution (40% PEG 8000, 1.2 M sodium chloride, and 1× phosphate-buffered saline; Sigma-Aldrich, Gillingham, UK) was added to the virus stock and incubated at 4°C overnight before centrifuging at 3,000 × *g* for 40 min. The resulting pellet was resuspended at 10× concentration in either DMEM or artificial saliva.

Artificial saliva was prepared according to ASTM E2721-16 (37), comprising 1.54 mM KH_2_PO_4_ (Sigma-Aldrich), 2.46 mM K_2_HPO_4_ (Fisher Scientific, Loughborough, UK), 0.04 mg/liter MgCl_2_·7H_2_O (Fisher Scientific), 0.11 g/liter NH_4_Cl (Sigma-Aldrich), 0.12 g/liter (NH_2_)_2_CO (Sigma-Aldrich), 0.13 g/liter CaCl_2_ (Sigma-Aldrich), 0.19 g/liter KSCN (Sigma-Aldrich), 0.42 g/liter NaHCO_3_ (Fisher Scientific), 0.88 g/liter NaCl (Fisher Scientific), 1.04 g/liter KCl (SLS, Wilford, UK), and 3 g/liter mucin (Sigma-Aldrich) at pH 7.

Virus stocks were stored at −80°C prior to use.

### Virus titration.

Virus suspensions were serially diluted in DMEM with 5% FBS and transferred onto BHK-21 (HCoV-OC43) or MRC-5 (HCoV-229E) cell monolayers seeded in a 96-well format. Plates were incubated (33°C, 5% CO_2_) for 4 days (HCoV-OC43) or 7 days (HCoV-229E) before scoring wells for cytopathic effect (CPE); the 50% tissue culture infectious dose (TCID_50_) was then calculated using the Karber method (38).

### Development of methodologies to recover coronaviruses from textiles.

**(i) Recovery media.** Sterile 25-cm^2^ swatches of 100% cotton were inoculated with 200 μl HCoV-OC43 (5 log_10_/25 cm^2^). After 5 min of contact, the swatches were placed in 5 ml DMEM (Lonza, Basel, Switzerland) with 5% FBS and 1% penicillin-streptomycin, PBS (Oxoid, Basingstoke, UK), or MRD (Oxoid, Basingstoke, UK) and vortexed for 1 min. Controls were the recovery medium with HCoV-OC43 alone (no-textile control) and no-virus controls (with and without textile). Supernatants were titrated on BHK-21 cells, and the percent recovery of HCoV-OC43 from cotton compared to the no-textile control was calculated.

**(ii) Agitation method.** Cotton swatches were inoculated with HCoV-OC43 as described above. After 5 min of contact, the swatches were placed in 5 ml PBS and either vortexed for 1 min, paddle blended using a Stomacher machine (Seward, Worthing, West Sussex, UK) at 230 rpm for 1 min, or vigorously shaken by hand 30 times. Controls were PBS with HCoV-OC43 alone (no-textile control) and no-virus controls (with and without textile). The supernatants were titrated on BHK-21 cells, and percent recovery was calculated.

### Stability of infectious HCoV-OC43 and HCoV-229E on textiles.

Sterile 25-cm^2^ swatches of 100% cotton, polycotton (65% cotton, 35% polyester), and 99.3% woven polyester were inoculated with 200 μl HCoV-OC43 or HCoV-229E (5 log_10_ TCID_50_/25 cm^2^) and incubated at room temperature (19.0 ± 0.5°C, 34% ± 2% relative humidity) in a class 2 cabinet. At 0, 2, 6, 18, 24, and 72 h postinoculation, infectious virus was recovered from the textile by shaking vigorously by hand 30 times in 5 ml PBS. No-virus controls (DMEM-inoculated textile samples) were also performed. Supernatants were titrated on BHK-21 (HCoV-OC43) or MRC-5 (HCoV-229E) cells, and viral titer was determined as described above.

Leaching of the viral inoculum from cotton and polycotton textile swatches onto the petri dish housing the swatch was investigated. Polyester was not absorbent and therefore was not tested. Sterile swatches of cotton and polycotton were inoculated with HCoV-OC43 as described above. After 0, 6, and 24 h of incubation at room temperature, the swatch was removed and the petri dish was swabbed thrice using a cotton swab. Swabs were vortexed for 30 s in 5 ml PBS, and the supernatant was titrated on BHK-21 cells.

### Wet/dry attachment of HCoV-OC43 to textile fibers.

Sterile 25-cm^2^ swatches of cotton, polycotton, and polyester were inoculated with 200 μl HCoV-OC43 (5 log_10_ TCID_50_/25 cm^2^). Identical swatches were first moistened with 200 μl PBS prior to inoculation with HCoV-OC43. The swatches were incubated at room temperature for 30 min to allow absorption into the textile. Infectious virus was recovered by two methods: shaking intact textile swatches by hand 30 times in 5 ml PBS and homogenizing (destroying) textile samples with sterile scissors prior to shaking by hand 30 times in PBS.

No-virus controls were included. The supernatants were titrated on BHK-21 cells, and TCID_50_ was calculated as described above. Comparisons of infectious virus recovery between intact and homogenized samples were used to infer HCoV-OC43 attachment to or entrapment by textile fibers.

### Transfer of HCoV-OC43 from textiles to other surfaces.

Sterile 25-cm^2^ swatches of cotton, polycotton, and polyester were inoculated with 200 μl HCoV-OC43 (5 log_10_ TCID_50_/25 cm^2^). Samples were incubated for 5 min to allow absorption of the inoculum or incubated at room temperature for the maximum time where HCoV-OC43 was detectable for each textile (polycotton, 2 h; cotton, 18 h; polyester, 72 h); inoculated textile swatches (donor swatches) were placed in contact with a sterile swatch of the same textile or a 25-cm^2^ swatch of polyvinyl chloride (PVC) polyurethane (PUR) safety flooring (Polyflor, Whitefield, UK) for 10 s under 100 g pressure (39). The transfer of infectious virus to textile was determined by shaking by hand 30 times in PBS. Infectious virus was recovered from PVC PUR by swabbing thrice with cotton swabs (SLS, Wilford, UK), vortexing for 30 s in 5 ml PBS, and filtering with a 0.45-μm polyether sulfone (PES) syringe filter (Fisher Scientific, Loughborough, UK). Supernatants were titrated on BHK-21 cells, and viral titer was determined as described above.

### Removal of HCoV-OC43 from textiles with domestic, industrial, and on-premises laundering processes.

**(i) Preparation of textile swatches.** Sterile 25-cm^2^ 100% cotton swatches were inoculated with 200 μl HCoV-OC43 (8 log_10_ TCID_50_/25 cm^2^) suspended in either DMEM or artificial saliva as an interfering substance and left to absorb at room temperature for 30 min before laundering. Identical swatches were shaken by hand 30 times in 5 ml PBS to determine the starting viral load present on the swatches.

**(ii) Domestic laundering cycle.** Inoculated swatches were placed in the drum of an Indesit IWSD61251 Eco machine along with two sterile swatches (to measure cross-contamination) and 2-kg polycotton makeweights (AATCC ballast type three; James Heal, Halifax, UK). The temperature was monitored during the wash process using an iButton Thermochron data logger (Measurement Systems, Newbury, UK). Washes were conducted at ambient temperature (23.44 ± 0.06°C), with or without 20 g standard ECE nonphosphate reference A detergent (40), and at 40°C with detergent.

Washed swatches were shaken by hand 30 times in 5 ml PBS, and the supernatant was titrated on BHK-21 cells. No-virus controls (culture medium- or artificial saliva-inoculated swatches) were performed throughout.

**(iii) Effect of domestic laundering in makeshift laundry bags.** Domestic washes were also performed as described above where test swatches were enclosed within a 50% polyester-50% cotton blend pillowcase to simulate the practice by nurses of placing their uniforms in a pillowcase or reusable bag for laundering.

**(iv) Industrial laundering cycles.** Inoculated cotton swatches, sterile cotton swatches, and 2-kg makeweights prepared as described above were laundered in a commercial washing machine (JLA, Ripponden, UK) using the simulated industrial laundering or OPL cycles described in Table 2. Washes were conducted without temperature and detergent (ambient water only) or with temperature and detergent. Ambient wash temperatures were 24.08 ± 0.07°C for industrial cycles and 22.52 ± 0.04°C for OPL cycles.

For industrial cycles, 2.5 ml/kg Power Extract (Christeyns, Ghent, Belgium) and 3 ml/kg Cool Care detergent (Christeyns, Ghent, Belgium) were added during the prewash stage and 16 ml/kg Cool Asepsis disinfectant (Christeyns, Ghent, Belgium) was added to the main wash stage. For OPL cycles, 3 ml/kg liquid detergent (Christeyns, Ghent, Belgium) was added to the prewash stage and a further 10 ml/kg detergent (Christeyns, Ghent, Belgium) was added to the main wash stage.

### Moist heat thermotolerance of HCoV-OC43.

A 200-μl aliquot of HCoV-OC43 (8 log_10_ TCID_50_/ml) was added to 5 ml sterile tap water and subjected to the temperature sequences of the domestic, industrial, or on-premises laundering processes described in Table 2 by heating and cooling in a water bath. Industrial wash parameters were additionally investigated with 50 and 60°C main wash stages. A control of HCoV-OC43 in water at ambient temperature for 1 h 35 min was included, and no-virus controls (200 μl DMEM in tap water) were performed. The test solutions were then titrated on BHK-21 cells, and viral titer was determined as described above.

### Statistical analysis.

All experiments were conducted in triplicate on separate occasions (*n* = 3), except wash tests, which were conducted as biological duplicates in three independent washes (*n* = 6). Viral quantification of each sample was performed with technical quadruplicates. The distribution of data was tested for normality using the Shapiro-Wilk test and homogeneity of variances using Levene’s test using SPSS version 26 (IBM, Armonk, NY, USA). The significance of differences was determined by one-way analysis of variance (ANOVA) with Tukey’s *post hoc* test where appropriate. Where assumptions of normality were violated, independent-sample Kruskal-Wallis tests with multiple comparisons or Mann-Whitney U tests were performed.
